# MOLGENIS Armadillo: a lightweight server for federated analysis using DataSHIELD

**DOI:** 10.1093/bioinformatics/btae726

**Published:** 2024-12-02

**Authors:** Tim Cadman, Mariska K Slofstra, Marije A van der Geest, Demetris Avraam, Tom R P Bishop, Tommy de Boer, Liesbeth Duijts, Sido Haakma, Eleanor Hyde, Vincent Jaddoe, Tarik Karramass, Fleur Kelpin, Yannick Marcon, Angela Pinot de Moira, Dick Postma, Clemens Tolboom, Ruben L Veenstra, Stuart Wheater, Marieke Welten, Rebecca C Wilson, Erik Zwart, Morris Swertz

**Affiliations:** Department of Genetics, Genomics Coordination Center, University Medical Center Groningen, University of Groningen, Groningen, 9700 RB, The Netherlands; Department of Genetics, Genomics Coordination Center, University Medical Center Groningen, University of Groningen, Groningen, 9700 RB, The Netherlands; Department of Genetics, Genomics Coordination Center, University Medical Center Groningen, University of Groningen, Groningen, 9700 RB, The Netherlands; Department of Public Health, University of Copenhagen, Copenhagen, 1353, Denmark; Medical Research Council Epidemiology Unit, University of Cambridge School of Clinical Medicine, Cambridge, CB2 0QQ, United Kingdom; Department of Genetics, Genomics Coordination Center, University Medical Center Groningen, University of Groningen, Groningen, 9700 RB, The Netherlands; Department of Neonatal and Pediatric Intensive Care, Division of Neonatology, Erasmus MC, University Medical Center Rotterdam, Rotterdam, 3015 GD, The Netherlands; Department of Pediatrics, Erasmus MC, University Medical Center Rotterdam, Rotterdam, 3015 GD, The Netherlands; Department of Genetics, Genomics Coordination Center, University Medical Center Groningen, University of Groningen, Groningen, 9700 RB, The Netherlands; Department of Genetics, Genomics Coordination Center, University Medical Center Groningen, University of Groningen, Groningen, 9700 RB, The Netherlands; Department of Pediatrics, Erasmus MC, University Medical Center Rotterdam, Rotterdam, 3015 GD, The Netherlands; Generation R Study Group, Erasmus MC, University Medical Center Rotterdam, Rotterdam, 3015 GD, The Netherlands; Department of Neonatal and Pediatric Intensive Care, Division of Neonatology, Erasmus MC, University Medical Center Rotterdam, Rotterdam, 3015 GD, The Netherlands; Department of Pediatrics, Erasmus MC, University Medical Center Rotterdam, Rotterdam, 3015 GD, The Netherlands; Department of Genetics, Genomics Coordination Center, University Medical Center Groningen, University of Groningen, Groningen, 9700 RB, The Netherlands; Epigeny, Paris, France; Department of Epidemiology and Biostatistics, Imperial College London, London, W2 1PG, United Kingdom; Department of Genetics, Genomics Coordination Center, University Medical Center Groningen, University of Groningen, Groningen, 9700 RB, The Netherlands; Department of Genetics, Genomics Coordination Center, University Medical Center Groningen, University of Groningen, Groningen, 9700 RB, The Netherlands; Department of Genetics, Genomics Coordination Center, University Medical Center Groningen, University of Groningen, Groningen, 9700 RB, The Netherlands; Arjuna Technologies, Newcastle Helix, Urban Science Building, Newcastle upon Tyne, NE4 5TG, United Kingdom; Department of Pediatrics, Erasmus MC, University Medical Center Rotterdam, Rotterdam, 3015 GD, The Netherlands; Generation R Study Group, Erasmus MC, University Medical Center Rotterdam, Rotterdam, 3015 GD, The Netherlands; Department of Public Health, Policy and Systems, University of Liverpool, Liverpool, L69 3GF, United Kingdom; Department of Genetics, Genomics Coordination Center, University Medical Center Groningen, University of Groningen, Groningen, 9700 RB, The Netherlands; Department of Genetics, Genomics Coordination Center, University Medical Center Groningen, University of Groningen, Groningen, 9700 RB, The Netherlands

## Abstract

**Summary:**

Extensive human health data from cohort studies, national registries, and biobanks can reveal lifecourse risk factors impacting health. Combining these sources offers increased statistical power, rare outcome detection, replication of findings, and extended study periods. Traditionally, this required data transfer to a central location or separate partner analyses with pooled summary statistics, posing ethical, legal, and time constraints. Federated analysis—which involves remote data analysis without sharing individual-level data—is a promising alternative. One promising solution is DataSHIELD (https://datashield.org/), an open-source R based implementation. To enable federated analysis, data owners need a user-friendly way to install the federated infrastructure and manage users and data. Here, we present MOLGENIS Armadillo: a lightweight server for federated analysis solutions such as DataSHIELD.

**Availability and implementation:**

Armadillo is implemented as a collection of three packages freely available under the open source licence LGPLv3: two R packages downloadable from the Comprehensive R Archive Network (CRAN) (“MolgenisArmadillo” and “DSMolgenisArmdillo”) and one Java application (“ArmadilloService”) as jar and docker images via Github (https://github.com/molgenis/molgenis-service-armadillo).

## 1 Background

Globally, a vast amount of data has now been collected on human health, including in cohort studies (Jaddoe *et al.* 2006), national registries (Schmidt *et al.* 2015), and biobanks (Bycroft *et al.* 2018). Analysis of this data has the potential to answer important research questions, particularly questions about lifecourse risk factors that affect human health and development. Whilst analysis of individual data sources is important, combining multiple data sources has a number of advantages. These include increased statistical power to explore exposure heterogeneity and detect rare outcomes, the potential to replicate findings in different settings (which may have different confounding structures) and an increase in the periods of lifecourse over which outcomes can be studied (Pinot de Moira *et al.* 2021, Cadman *et al.* 2024a).

Traditionally, analysis using multiple data sources was conducted by either transferring the individual-level data to a central location or having each partner conduct the analyses separately, followed by pooling of summary statistics centrally. Both methods have advantages and drawbacks. Transferring data to a central location makes the analysis easier because it can be conducted (and iterated) by one researcher, however there can be ethico-legal constraints on data transfer (Knoppers *et al.* 2011). By contrast, no data transfer is required when partners conduct separate analyses and send results to a central location for meta-analysis. However, conducting the analysis can be difficult as errors need to be debugged by each partner, and analysis must be re-run by each partner when there are changes or revisions.

Federated analysis—where data is analyzed remotely without sharing individual-level data—is a promising alternative (Doiron *et al.* 2013). It allows remote analysis whilst allowing the data owner to retain control of their data. Federated approaches work by sharing only model parameters or summary statistics which are not disclosive. They exist on a spectrum between (i) “hub-and-spokes” approaches, where some central infrastructure is required to coordinate the communication between the client and the servers, or (ii) “peer-to-peer” approaches, where a user sends an analysis request to a node, and this node then sends queries to other nodes in the network. Implementations also vary in whether they permit interactive querying of data or batch-running of scripts/commands (Dursi *et al.* 2021).

One mature implementation of federated analysis is DataSHIELD, an open-source R-based tool. DataSHIELD is a hub-and-spoke approach, which allows researchers to send analysis commands from the client-side to remote data sources (server-side), and receive only non-disclosive summary statistics in return. DataSHIELD mitigates disclosure risk by preventing users from viewing, transferring or copying individual-level data (Gaye *et al.* 2014).

Federated analysis using DataSHIELD has been a key component of several international multi-centre projects. DataSHIELD is an apt solution for epidemiological research as it allows the interactive manipulation and summarizing of data, which despite harmonization can still be required to prepare data and conduct analysis. Recent projects using DataSHIELD include four H2020 and Horizon Europe funded projects aiming to establish a network of pregnancy and childhood cohorts and provide a federated analysis infrastructure: LifeCycle (Jaddoe *et al.* 2020, Pinot de Moira *et al.* 2021) (researching the role of novel integrated markers of early-life stressors on health across the lifecycle), ATHLETE (understanding and preventing health effects of environmental hazards and their mixtures) (Vrijheid *et al.* 2021), LongITools (studying the interactions between environmental, lifestyle and biological factors to determine people’s risks of developing cardiometabolic non-communicable disease), and EUCAN-connect (a cross-project infrastructure initiative including LifeCycle, ATHLETE and LongITools).

One of the key tasks for partners within these projects has been to install and maintain servers and upload harmonised data so that they can be analyzed remotely to address scientific research questions. To work effectively, each cohort needs a server set-up that: (i) has low resource use and is scalable so it remains stable with large datasets, (ii) is operationally quick and (iii) is user-friendly. During the LifeCycle project, the importance of a user-friendly solution became clear, as many of the data managers in participating cohorts had a research background rather than an IT background.

To meet the need for such a server set-up, we reviewed the existing options. The only previous option was Opal, which is an open-source software package providing functionality for data management, harmonization and dissemination (Doiron *et al.* 2017). However, we noted that Opal had many rich features that were superfluous to our current needs, and it increased server requirements and administration. Our aim therefore was to create a light-weight, user-friendly server (MOLGENIS “Armadillo”) that implements a DataSHIELD federated analysis environment.

## 2 Features

Armadillo performs three main operations: (i) data management, (ii) permission management, and (iii) profile management. These are manageable either via a user interface (UI) or through a companion R package (MolgenisArmadillo). Technical monitoring is also available through the UI. Screenshots of the UI are included in Supplementary Materials.

### 2.1 Data management

While Armadillo can handle any file type, we use parquet files to store tabular data as they allow for efficient storage (Vohra and Vohra 2016). Files are stored on the file system rather than a database to enable lightweight implementation. Armadillo also supports resources such as OMICS and exposome resources via the R package Resourcer (Marcon *et al.* 2021). Data are organized into projects (folders). Data managers can upload data via either the UI or the companion R package MolgenisArmadillo. For ease of use, when uploading via R, RDA/RData files are automatically converted to parquet format. Previews of the uploaded data are also available within the UI.

To increase data security, researchers are normally only given access to the subset of variables specified in the signed Data Use Agreement (DUA) that is required by each participating cohort or study, for which approval for that specific cohort or study from local institutional review boards is available, including written informed consent for using participant data and the possibility to exchange original participant data. Data managers can create these subsets in the UI by selecting variables from a larger data frame or via the R package. Using the UI may be easier for small tasks, but the R interface is generally more efficient as it allows required variables for multiple tables to be specified in CSV files, which are then imported. Creating subsets via R is also reproducible as the script can be saved.

### 2.2 Permission management

To access project data stored on an Armadillo server, researchers first need to complete the required data governance procedure, which normally includes signing a DUA. Once approval is in place, researchers need to register through an authentication server (e.g. using FusionAuth or Keycloak) via OpenID Connect (such as LifeSciences AAI). Using the UI, the data manager can then add the researcher as a user and grant them access permissions to their specific project.

### 2.3 Profile management

For any R function to be available in DataSHIELD, it needs to be implemented in a DataSHIELD-specific package. This is in order to translate it into a client-server format, and write bespoke checks to ensure that any output will not disclose individual-level data. Many DataSHIELD R packages are now available, providing functionality for different types of analysis (https://wiki.datashield.org/en/opmanag/community_packages). For these to be available to the researcher, they need to be installed on the Armadillo server. To streamline this process, in conjunction with the DataSHIELD community, we created “profiles,” a collection of named and versioned analysis packages stored within Docker containers (https://wiki.datashield.org/en/opmanag/standard-profiles-and-platforms). The specific packages included within profiles are determined by the needs of consortia, and each consortium can choose an existing profile or devise its own. Installing a profile installs all of the bundled DataSHIELD packages.

Profiles are managed within the UI. Admins can add, remove and manage profiles; whitelist specific R packages; blacklist specific functions; start and stop the Docker containers and set disclosure controls. This ensures that admins retain control over exactly which functionality is available to the user, which may vary depending on the data type, context and legal framework within a country or region.

### 2.4 Technical monitoring

An additional “insight” section in the UI provides access to the log files (e.g. to view errors) and server metrics, providing insight into the stability of the application and the resources available on the server. This was implemented to provide admins access to technical information without needing to use the command line, potentially allowing issues to be resolved faster. The audit log also provides a complete trail of actions with the data so that admins can monitor and review potentially inappropriate actions.

## 3 Armadillo in the wild

We have successfully deployed Armadillo in several research networks for multiple data owners using the following flow. First, Armadillo is installed on one or more servers at participating institutions, and these servers are placed behind a firewall. As an added layer of security, we have implemented a Central Analysis Server (CAS). The CAS contains an R Studio environment with pre-installed client-side DataSHIELD packages on which users can conduct analyses. The IP address of the CAS is whitelisted on each Armadillo server, which means that researchers can only access Armadillo by logging in to the CAS. This also means only the CAS needs whitelisting, rather than whitelisting the IP addresses of individual researchers, which keeps the user management in large consortia maintainable. Next, data is uploaded onto each server. Researchers then follow the data access procedures of each cohort they wish to include in their project, typically including the arrangement of a DUA as described above. Once logged in, users are able to assign data for the project(s) to which they have received access and begin their analysis. This set-up has been successfully used throughout the LifeCycle and ATHLETE projects, resulting in a number of scientific publications (e.g. Torres Toda *et al.* 2022, Vinther *et al.* 2023, Cadman *et al.* 2024b, Guerlich *et al.* 2024).

## 4 Technical implementation

Armadillo is implemented via three packages: (i) ArmadilloService (Java backend and admin user interface), (ii) MolgenisArmadillo (Admin R client), and (iii) DSMolgenisArmadillo (Armadillo R implementation of DataSHIELD). The backend consists of three main components used to manage: (i) data storage and APIs (Application Programming Interfaces), (ii) the connection with DataSHIELD and execution of R code in Java, and (iii) the UI (Table 1).

**Table 1.** Key terms used in manuscript.

Term	Explanation
R	Statistical software environment
Federated analysis	Remote analysis on distributed servers
DataSHIELD	Federated analysis software
ArmadilloService	Java backend
MolgenisArmadillo	Admin R client
DSMolgenisArmadillo	Armadillo DataSHIELD implementation

**Figure 1.**
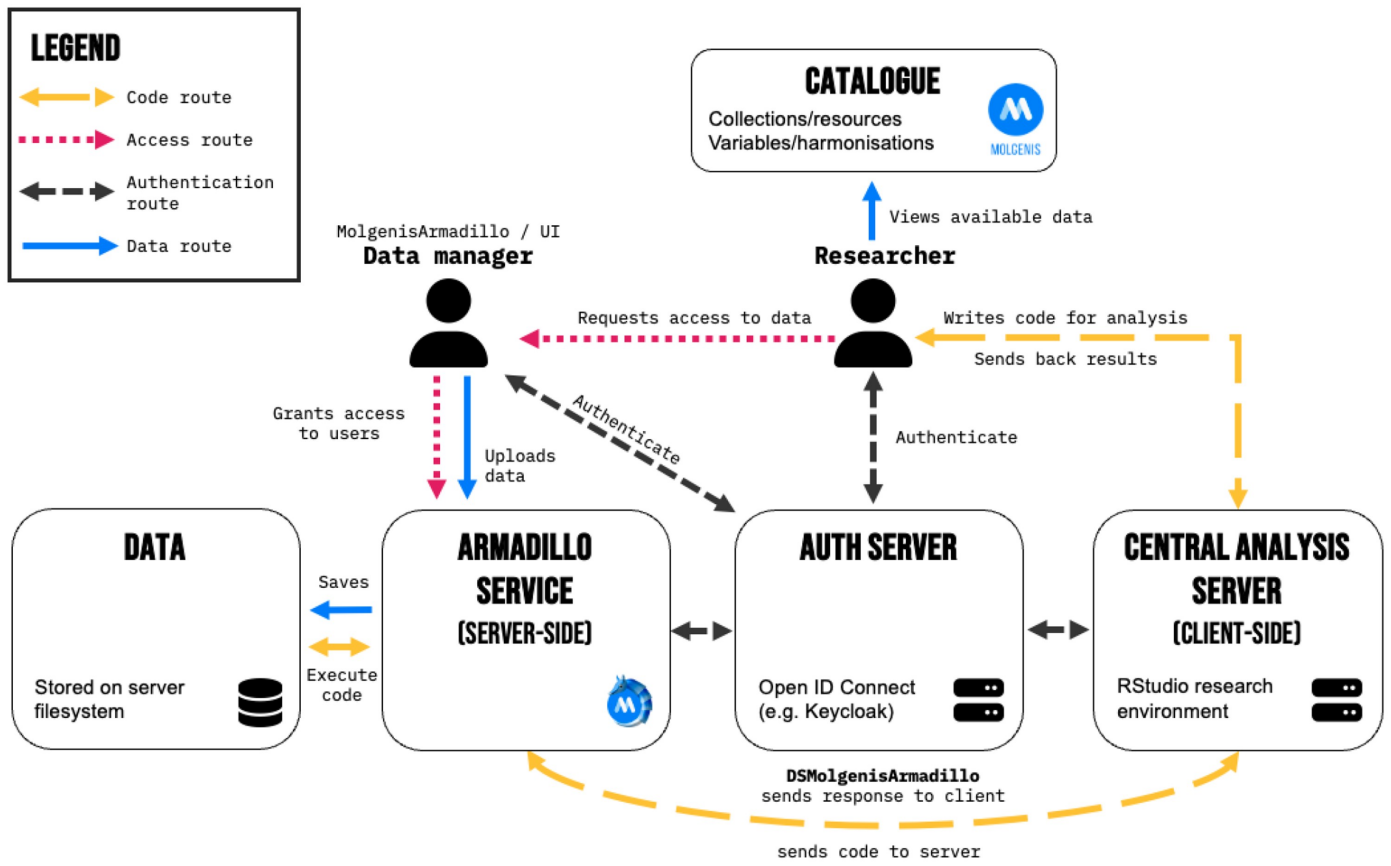
Armadillo information flow.

To understand the implementation of Armadillo, it is useful to have a general understanding of how DataSHIELD works. Each DataSHIELD implementation consists of two R packages: (i) a “client-side package” that is loaded locally within the analyst’s R session and (ii) a “server-side” package that is installed and loaded on the Armadillo server. First, users create a session using the R functions from the client-side DataSHIELD packages. At the researcher’s request, analysis methods in the client-side packages send commands to each server. After checks to determine that the server-side method is permitted for that analyst and will not cause a disclosure based on argument alone, a DataSHIELD server-side function is invoked. In the case of assign-type functions, data is stored on the server and nothing is returned. In the case of aggregate functions, after a number of disclosure checks are performed, non-disclosing summary statistics are returned. The information flow is displayed in Fig. 1.

ArmadilloService is a Java application written using the Spring Boot framework (version 3) that runs on a Linux-based server. Research data is stored on the file system of the server where Armadillo is installed. Data managers can upload their data to the server using either the REST API, the UI, or R package MolgenisArmadillo.

When an analysis command is sent from a DataSHIELD client-side package, DSMolgenisArmadillo connects to the API, which executes the DataSHIELD server-side R code. If executed correctly, the server will either create an object on the server (for DataSHIELD assign functions) or return non-disclosive summary statistics (for DataSHIELD aggregate functions). If the command returns an error, this will also be returned to the user via the API. If the user has created objects on Armadillo (e.g. transformed variables) within a particular session, an R workspace can be saved on the server side. Since this saved workspace contains individual participant data, it cannot be viewed directly and can only be queried by permitted non-disclosing DataSHIELD functions. This workspace can also be restored at the beginning of a new session.

The UI is built using the Vue JS framework. It is currently made solely for data management purposes and is therefore only available to administrators. All views in the UI (projects, users, profiles, insight) have their own component. Additional components for generic functionality were created so that features can be easily added and extended. Bootstrap classes and icons were used for styling throughout the application to maintain a consistent appearance across all views. Communication with the Armadillo backend happens through the Armadillo REST API (Swagger documentation included). Most components and methods are covered by unit tests to ensure the application’s quality.

## 5 How to get Armadillo

Armadillo is maintained as part of the long running http://molgenis.org for scientific software at https://github.com/molgenis/molgenis-service-armadillo, with the client maintained at https://github.com/molgenis/molgenis-r-armadillo under free and open source licences LGPLv3. All packages are available on the The Comprehensive R Archive Network (CRAN).

## 6 Future directions

We are continuously developing Armadillo and are currently working on new features to overcome challenges we have encountered. One challenge has been providing users and data-managers with real-time information on the status of the network. To this end, we are developing a system to monitor Armadillo nodes and provide information on server status, DataSHIELD package availability and other metrics. As analysis with DataSHIELD is currently computationally slower than running R locally, we are working to improve performance. We are also streamlining the installation of Armadillo DataSHIELD profiles to allow more rapid updates. Finally, whilst Armadillo is currently implemented for DataSHIELD, there are other federated analysis solutions. A long-term goal is to implement Armadillo for other platforms such as Vantage6 (Moncada-Torres *et al.* 2020).

## Supplementary Material

btae726_Supplementary_Data
