# Supplementary material for: MOLGENIS Armadillo: a lightweight server for federated analysis using DataSHIELD
Source: Bioinformatics. 2024 Dec 2;41(1):btae726. doi: 10.1093/bioinformatics/btae726 (PMC11734753; doi:10.1093/bioinformatics/btae726)
Supplement: btae726_Supplementary_Data [file btae726_supplementary_data.docx]

Supplementary Materials

**Supplementary Figure 1: Project View**

**
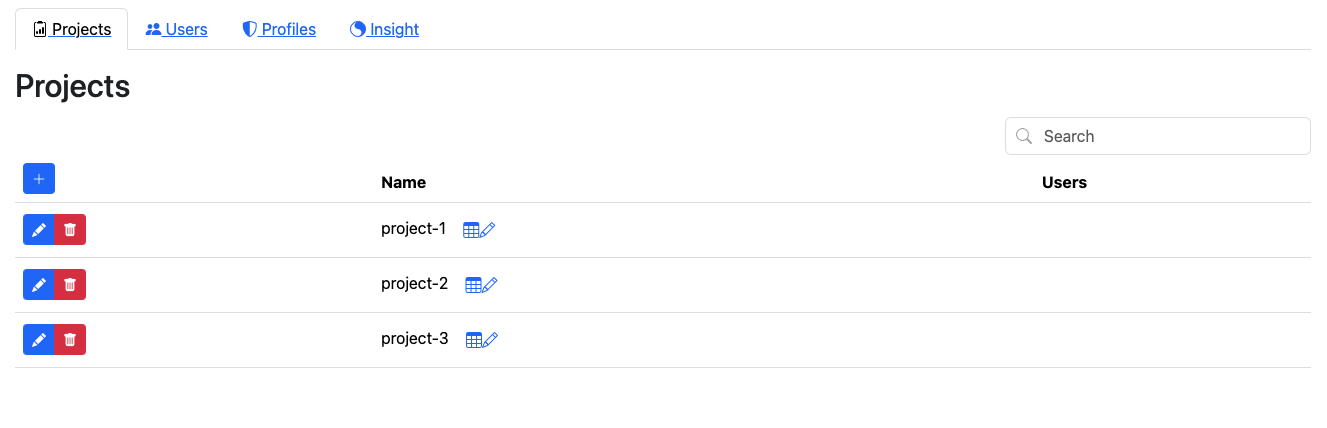
**

**Supplementary Figure 2: Users view**

**
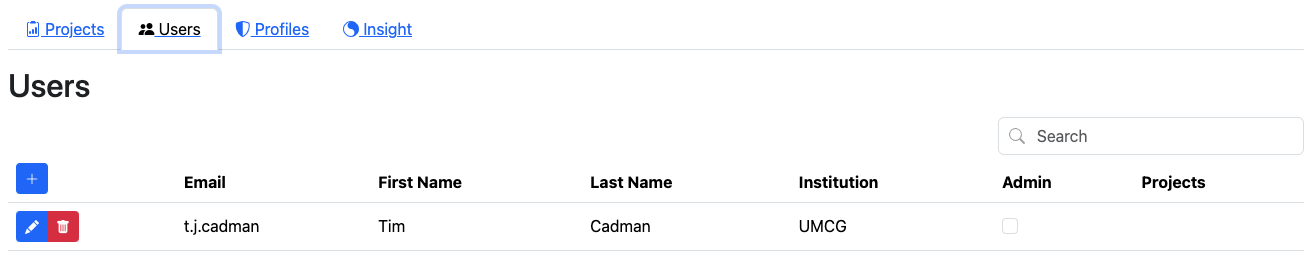
**

**Supplementary Figure 3: Profiles view**

**
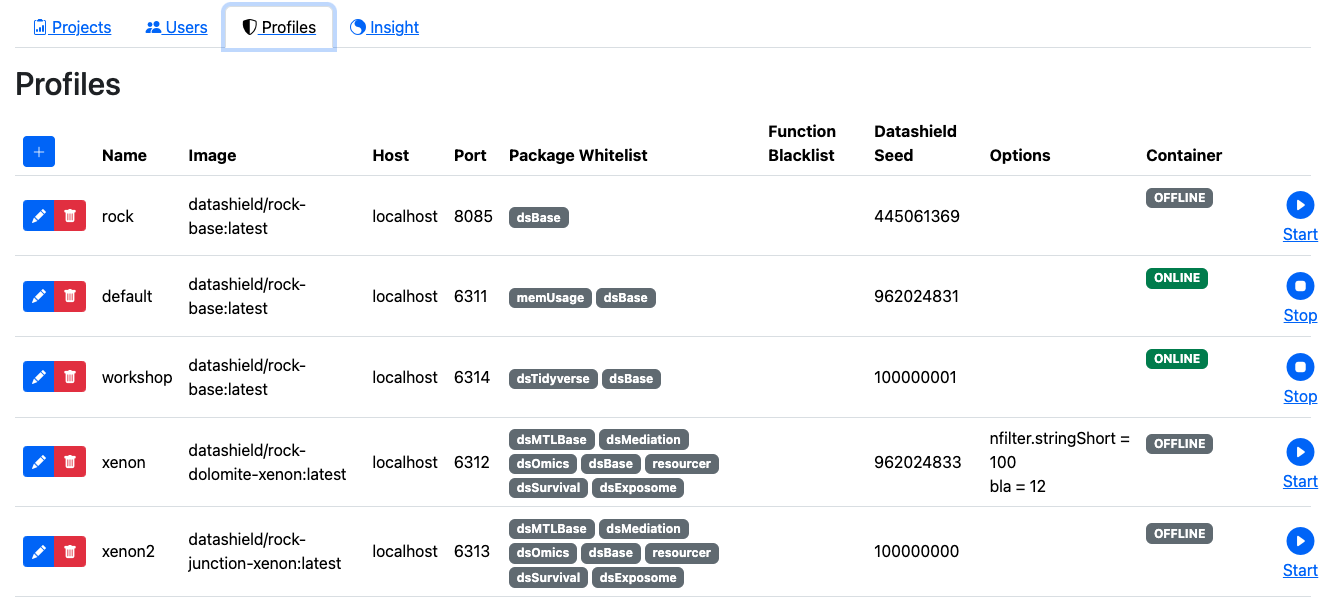
**
